# Sialyl Residues Modulate LPS-Mediated Signaling through the Toll-Like Receptor 4 Complex

**DOI:** 10.1371/journal.pone.0032359

**Published:** 2012-04-09

**Authors:** Chiguang Feng, Nicholas M. Stamatos, Anatoliy I. Dragan, Andrei Medvedev, Melissa Whitford, Lei Zhang, Chang Song, Prasad Rallabhandi, Leah Cole, Quan M. Nhu, Stefanie N. Vogel, Chris D. Geddes, Alan S. Cross

**Affiliations:** 1 Center for Vaccine Development, University of Maryland School of Medicine, Baltimore, Maryland, United States of America; 2 Institute of Human Virology and Department of Medicine, University of Maryland School of Medicine, Baltimore, Maryland, United States of America; 3 Institute of Fluorescence, University of Maryland, Baltimore County, Baltimore, Maryland, United States of America; 4 Department of Microbiology and Immunology, University of Maryland School of Medicine, Baltimore, Maryland, United States of America; University Paris Sud, France

## Abstract

We previously reported that neuraminidase (NA) pretreatment of human PBMCs markedly increased their cytokine response to lipopolysaccharide (LPS). To study the mechanisms by which this occurs, we transfected HEK293T cells with plasmids encoding TLR4, CD14, and MD2 (three components of the LPS receptor complex), as well as a NFκB luciferase reporting system. Both TLR4 and MD2 encoded by the plasmids are α-2,6 sialylated. HEK293T cells transfected with TLR4/MD2/CD14 responded robustly to the addition of LPS; however, omission of the MD2 plasmid abrogated this response. Addition of culture supernatants from MD2 (sMD2)-transfected HEK293T cells, but not recombinant, non-glycosylated MD2 reconstituted this response. NA treatment of sMD2 enhanced the LPS response as did NA treatment of the TLR4/CD14-transfected cell supplemented with untreated sMD2, but optimal LPS-initiated responses were observed with NA-treated TLR4/CD14-transfected cells supplemented with NA-treated sMD2. We hypothesized that removal of negatively charged sialyl residues from glycans on the TLR4 complex would hasten the dimerization of TLR4 monomers required for signaling. Co-transfection of HEK293T cells with separate plasmids encoding either YFP- or FLAG-tagged TLR4, followed by treatment with NA and stimulation with LPS, led to an earlier and more robust time-dependent dimerization of TLR4 monomers on co-immunoprecipitation, compared to untreated cells. These findings were confirmed by fluorescence resonance energy transfer (FRET) analysis. Overexpression of human Neu1 increased LPS-initiated TLR4-mediated NFκB activation and a NA inhibitor suppressed its activation. We conclude that (1) sialyl residues on TLR4 modulate LPS responsiveness, perhaps by facilitating clustering of the homodimers, and that (2) sialic acid, and perhaps other glycosyl species, regulate MD2 activity required for LPS-mediated signaling. We speculate that endogenous sialidase activity mobilized during cell activation may play a role in this regulation.

## Introduction

Eukaryotic hosts can distinguish between “self” antigens and potential exogenous or endogenous “danger signals” through the expression of pattern recognition receptors (PRRs) on their cell surface and/or within the cell cytosol. Among the families of PRRs, the Toll-like receptors (TLRs) are the most well studied, with 10 distinct TLRs expressed on human cells and 12 on murine cells [1]. Poltarak et al identified TLR4 as the receptor for Gram-negative bacterial lipopolysaccharide (LPS), thereby characterizing the basis for the previously recognized resistance of C3H/HeJ and C57BL6/ScN mice to LPS lethality [2]. In the absence of a functional TLR4, these mice are unable to recognize LPS or to initiate a pro-inflammatory response. While the failure to recognize and initiate an intracellular signaling cascade may protect mice from lethal LPS intoxication, we and others have shown that such a signaling defect makes these mice highly susceptible to lethal bacterial infection [3], [4]. Thus, TLR4-initiated responses are required for host defenses against live, replicating bacterial infection, but may lead to deleterious responses as well. Subsequent studies have clearly established that while TLR4 recognizes LPS, it requires the presence of an adapter protein, MD-2, and an accessory molecule, CD14, for its function [5]. These three proteins form the TLR4 receptor complex. Further, each of these proteins must be glycosylated for their proper function [6], but neither the required carbohydrate moieties nor a putative mechanism for their regulation has been identified.

Glycosylation of molecules on the cell surface regulates their interaction with ligands, such as insulin, and with other glycoconjugates on the cell surface as well as determines the ability of the cell to interact with other cells during inflammation [7] . In part through its negative charge, terminal sialyl residues may modulate these molecular and cellular interactions [7]–[10]. Our laboratory has studied the role of sialic acid modulation in innate immune function. We previously demonstrated that sialidase activity in human polymorphonuclear leukocytes (PMN) resides within intracellular compartments, primarily secondary granules, and upon PMN activation, the sialidase activity translocates to the PMN surface where it becomes an integral membrane protein [11]. This enzyme may then remove sialyl residues from glycoconjugates on its own (*i.e.* autocrine) or adjacent (paracrine) cells. These cells become more motile, adhesive and more responsive to subsequent agonist stimulation [12]. Inhibition of PMN sialidase activity inhibits their recruitment to inflammatory sites in response to IL-8 *in vivo* and their transendothelial migration to chemokines *in vitro* [13]. Once at an inflammatory site, the removal of sialyl residues on the PMN β2 integrin and endothelial cell ICAM-1 exposes activation epitopes on each molecule that enhances their binding interaction [14]. In addition, restoration of sialylation on the PMN surface by a PMN sialyltransferase restores the non-activated phenotype [15]. Thus, the PMN can orchestrate its activation phenotype by modulating the sialylation at its cell surface through the activity of endogenous sialidase and sialyltransferase activities.

Modulation of sialic acid content on the surface of human peripheral blood mononuclear cells (PBMC) also affects their immune capacity. Pretreatment of PBMCs with an exogenous sialidase (*Clostridium perfringens*) markedly enhances the subsequent production of cytokines in response to LPS [16]. Inhibition of endogenous sialidase activity in monocyte-derived dendritic cells decreases LPS-induced cytokine production [17]. Since the TLR4 complex is the principal PRR for LPS, we reasoned that TLR4 is sialylated, and that removal of sialyl residues facilitates the recognition of LPS by this receptor complex and/or enhances subsequent downstream signaling. In the present studies, we demonstrate that not only TLR4 but also MD-2 is sialylated, and that removal of sialyl residues on each of these molecules enhances the functional response of cells to LPS.

## Materials and Methods

### Cell line and reagents

HEK293T human embryonic kidney cells (American Type Culture Collection) were cultured in DMEM (Invitrogen, Carlsbad, CA) supplemented with 10% FBS, 2 mM L-glutamine, 100 U/ml penicillin, and 100 µg/ml streptomycin all from Invitrogen. LPS (*E. coli* O111B4) was purchased from List Biologics (Campbell, CA). *C. perfringens* neuraminidase (Type X), anti-FLAG (MD2) antibody, and protein A agarose was purchased from Sigma (St. Louis, MO). The neuraminidase inhibitor, 2,3-Dehydro-2-deoxy-N-acetyl neuraminic acid (2-DN) and a control of similar MW and charge, 2-keto-3-deoxyoctulonic acid (KDO), were purchased from Calbiochem (Gibbstown, NJ). Anti-GFP was purchased from Invitrogen. TurboFect transfection reagent was purchased from Fermentas (Glen Burnie, MD); SuperFect transfection reagent was purchased from Qiagen (Valencia, CA). Recombinant human MD-2, recombinant human TLR4/MD2 complex, each fused to a polyhistidine tag, and recombinant human CD14 were purchased from R&D Systems, Inc. (Minneapolis MN)

### Plasmid construction

Except for pcDNA-TLR4-CFP, all expression plasmids were constructed as previously reported [18] and were prepared using EndoFree Plasmid Maxi kit (Qiagen). To construct the pcDNA-TLR4-CFP plasmid, the YFP fragment was excised from pcDNA-TLR4-YFP and replaced with CFP, which was isolated from pcDNA3-CFP (Addgene, Cambridge, MA) after restriction enzyme digestion. TLR4 glycosylation mutants have been generated by introducing the respective mutations N35A, N173A, N205A or their combinations, into pcDNA3-TLR4-YFP expression plasmid by PCR-based site-directed mutagenesis, as described [19]–[21], using the following primers: N35A, 5′-TGCGTGGAGGTGGTTCCTGCTATTACTTATCAAT GCATGGAGCTG-3′; N173A; 5′-TCAAATTACCTGAGTATTTTTCTGC TCTGACC AATCTAGAGCACTTGG-3′; N205A, 5′-GTTCTACATCAAATGCCCCTACTCGCTCTCTCTTTAGACCTGTCCCTG-3′. pcDNA3-TLR4-YFP P416A expression plasmid has been described [22].

Nucleotide sequencing (Genomic and Biopolymer Core Facility, University of Maryland, Baltimore, MD) of each construct was carried out to confirm the specific and correct introduction of the mutations and the absence of PCR-introduced errors.

### Transfection and Reporter assay

Transfection and reporter assays were performed as described previously [18]. Briefly, HEK293T cells were seeded onto 24-well plates (2×10^5^ cells/well) and incubated overnight. The cells were co-transfected with optimized amounts of plasmid mixture containing pcDNA3-TLR4-YFP, pcDNA3-huCD14, pEFBOS-MD2-FLAG, pELAM-luc (NF-kB Luciferase reporter), and pcDNA-renilla. After 20 h of recovery, cells were stimulated with LPS at the indicated concentrations overnight, and lysed in 1× reporter assay lysis buffer (Promega, Madison, WI). Firefly and renilla luciferase activities were assayed at LMax II^384^ microplate reader (Molecular Devices, Sunnyvale, CA) with injection of 100 µl of substrate from Luciferase Assay System or Renilla Luciferase Assay System individually (Promega). “Relative luciferase activity” was calculated by normalizing each sample's firefly luciferase activity to constitutive renilla luciferase activity measured within the same sample, and was represented as relative luciferase units (RLU). In some experiments, 2-DN (250 ug/ml) or KDO (250 ug/ml) were added to the medium during culture.

In reconstitution cultures, HEK293T cells were co-transfected with the above plasmid mixture without pcDNA3-MD2-FLAG, and supplemented with supernatant (SNT) from MD2-transfected HEK293T cell before LPS stimulation, as previously described [23]. For desialylation, cells were treated with *C. perfringens* neuraminidase (NA) (30 mU/ml) for 1 hour in PBS before LPS stimulation. SNT from MD2-transfected cells (sMD2) was treated with NA-agarose beads (Sigma) for one hour and separated with centrifugation. PBS and/or heat-inactivated NA were used as controls. For heat inactivation, NA or NA agarose was boiled for 30 minutes. Complete inactivation was confirmed in a sialidase activity assay.

### Transfection and Immunoprecipitation

HEK293T cells were seeded into 90-mm cell culture dishes at 10^6^ cells/mL in 5 mL of complete media, and incubated at 37°C in 5% CO_2_ overnight. Cells were transfected with 10 µg of plasmid mixture using TurboFect (Fermentas) or SuperFect (Qiagen) transfection reagent according to the manufacturer's recommendation. Complete media was replaced after 4–6 h. Cells were scraped and harvested 72 h after transfection, washed in HBSS and lysed using 1× Lysis Buffer (Cell Signaling Technology) containing protease inhibitors (Roche).

The cell lysates containing the proteins of interest were incubated with pre-cleaned Protein A agarose beads (Sigma) for 2 h at 4°C on a roller. Samples were spun down and anti-GFP antibody (Invitrogen) or anti-FLAG antibody (Sigma) was added to the supernatants (2 µg/mL) and incubated at 4°C for 4–6 h. Freshly cleared Protein A agarose beads were then added to each sample for an overnight incubation at 4°C on roller. Agarose beads were thoroughly washed with cold Lysis Buffer and re-suspended in 2× Laemmli's Sample Buffer. The immunoprecipitated protein was released after boiling for ten minutes and briefly centrifuged at 14,000 rpm.

### Western and lectin blots

The immunoprecipitated proteins were separated on SDS-PAGE gels (4–15% Tris-HCl, Bio-Rad) and transferred to 0.45 µm PVDF membranes. , The membranes were blocked with 3% BSA in TBS overnight and blotted with biotinylated *Sambucus nigra* lectin (SNA) or *Maackia amurensis* II lectin (MAAII) (Vector Laboratory) in 1% BSA in TBS for 1 h, re-blocked for 1 h, then incubated with streptavidin-HRP in 0.5% BSA in TBS. The membranes were washed three times in TBST (TBS containing 0.1% Tween20) between each described step. SuperSignal West Pico Chemiluminescent substrate (Thermo Scientific) was added to the membranes for 5 minutes prior to exposing them to film (Kodak). In some cases, the chemiluminescent intensity was detected by a Fujifilm CCD camera. Fetuin (Sigma) was used as a positive control and asialofetuin (Sigma) as a negative control for the lectin binding. Lectin SNA recognizes terminal sialic acids with an α-2,6 glycosidic linkage. Soluble sialic acids block its binding and abolished the bands (data not shown), which suggested the specificity of SNA binding.

### Fluorescence resonance energy transfer (FRET) assay

We analyzed the LPS-stimulated association of the TLR-4 receptors by FRET, using CFP or YFP-tagged TLR4 monomers. The fluorescence spectrum of the CFP protein overlaps the absorption spectrum of YFP, which makes them a donor-acceptor pair suitable for estimation of the change in distance between individual monomers [24], [25]. HEK293T cells were transfected with a mixture of plasmids encoding TLR4-YFP, TLR4-CFP, CD14, and MD2 and harvested 48 hours later. For NA treatment, the cells were incubated with 30 mU/ml of NA in PBS at 37°C for 1 hr. After washing, the cells were resuspended in PBS containing 100 µg/ml of cytidine monophosphate to inhibit resialylation by sialyltransferase. The cells then were transferred to a cuvette and stimulated with 10 µg/ml of LPS. Emission spectra of the fluorescent proteins (CFP and YFP) in living cells was measured every 5 minutes using a FluoroMax 4 spectrofluorimeter (Horiba Jobin Yvon, USA) equipped with a temperature-controlled cell holder (PeltierThermostabilization System F-3004). All experiments were undertaken at 37°C. The concentration of cells in all experiments was 2.5×10^6^ /ml. Samples were gently shaken in a cuvette to get homogenous cell distribution before recording the emission. The fluorescence was excited at 440 nm wavelength at which both fluorescent proteins (CFP and YFP) absorb light. To excite YFP protein fluorescence alone we used the 490 nm wavelength at which only YFP absorbs light. The fluorescence spectra of the transfected cells were recorded in the spectral range from 460 nm to 650 nm. The contribution of light scattering to the fluorescence spectra of transfected cells (background) was measured using samples containing the non-transfected HEK293T cells and those values were subtracted from the fluorescence signals of the sample (Figure S1A).

The FRET effect in HEK293T cells containing CFP- and YFP-tagged TLR4 proteins was analyzed using the ratio of fluorescence intensities measured at two wavelengths: 528 nm and 475 nm (fluorescence maximum of YFP and CFP, respectively) with excitation undertaken at 440 nm. The ratio F(528)/F(475) is sensitive to FRET due to strong enhancement of YFP (acceptor) fluorescence upon energy migration. This FRET ratio was calculated and plotted against time after stimulation cells with LPS.

### Adenovirus construction

The human *neu*1 or *neu*3 sequences were synthesized by PrimmBiotech (Cambridge, MA) using sequences deposited in GenBank (NM_000433.3 and NM_006656.5). The Ad-Neu1, Ad-Neu3, and control Ad-GFP viruses were generated using AdEasy Adenoviral Vector System (Stratagene, La Jolla, CA) according to the manufacturer's recommendation. Briefly, the *neu*1 and *neu*3 genes were inserted into a pShuttle-IRES-hrGFP-1 vector using restriction enzyme digestion and ligation. The *neu*1, *neu*3 inserted or empty shuttle vector was linearized and co-tranformed with pAdEasy-1 vector into BJ5183 competent cells to generate recombinant plasmid adeno-neu1, adeno-neu3, or adeno-GFP. The recombinant plasmids were extracted, and confirmed by Pac I digestion followed by agarose gel analysis. The plasmids with the correct recombination by size were further transformed into XL10-Gold cells for amplification. Sufficient amount of plasmids were tranfected into AD-293 cells using Lipofectamine (Invitrogen) for virus packaging. The primary virus stocks were further amplified in AD-293 cells and their titers quantified with plaque assay.

### Sialidase activity assay

Sialidase activity was assayed by using 2′-(4-methylumbelliferyl)-α-D-N-acetylneuraminic acid sodium salt hydrate (4-MUNANA, Sigma) as the substrate. The lysates from transfected cells were suspended in 200 µl of 50 mM sodium acetate buffer (pH 4.4), containing 0.1% Triton X-100 and Protease Inhibitor Cocktail (Sigma), and 25 µl of 4-MUNANA (2 mM) (Sigma). The mixtures were briefly vortexed and incubated for 1 hour at 37°C. The reaction was terminated with 1 ml of glycine buffer (pH 10.3) containing 0.133 M glycine, 60 mM NaCl and 42 mM Na_2_CO_3_. After the mixtures were centrifuged, the supernatants were collected and dispended into 96 well microplate (Costar) to measure the fluorescence intensity by Fluoroskan Ascent FL (Thermo Electron Corporation). The amount of free 4-Mu in each samples were interpolated with the intensities from a serial dilution of known concentration of 4-Mu (Sigma) using GraphPad Prism 4 (GraphPad software, Inc. La Jolla, CA).

### Statistical analysis

Statistical significance was calculated using Student's t test in Microsoft Excel; p values smaller than 0.05 were considered significant.

## Results

### 1. Desialylation enhances LPS-induced NFκB signaling in HEK293T cells transfected with TLR4 complex

**Figure 1:**
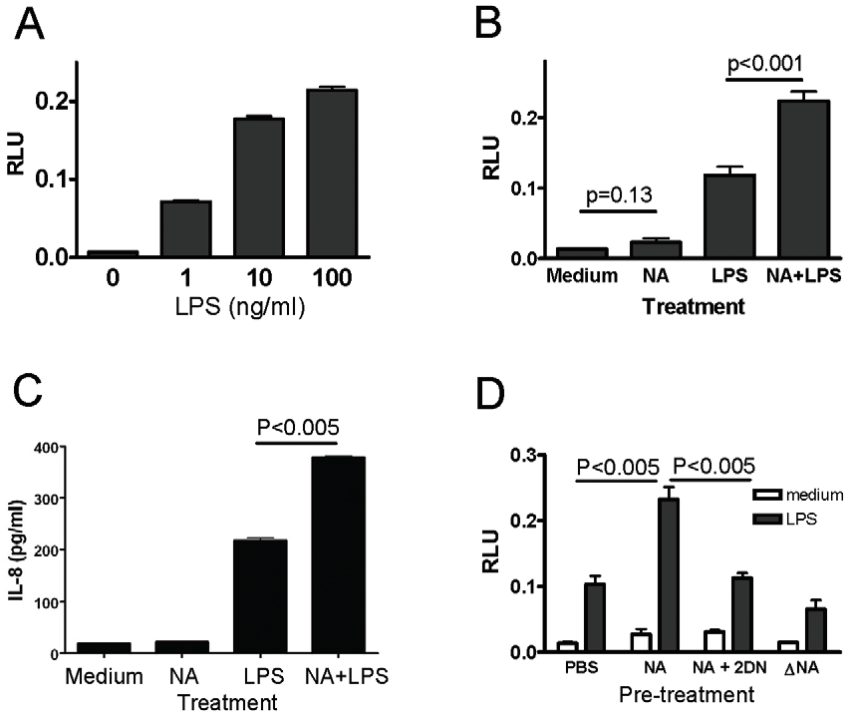
Neuraminidase treatment enhances cell response to LPS stimulation. HEK293T cells were transfected with a mixture of expression vectors encoding TLR4-YFP, MD2-FLAG, CD14, and firefly and Renilla luciferase genes. After 48 h, the transfected cells were stimulated with LPS (1 to 10 ng/ml) for 16 h and firefly vs. renilla luciferase activities were measured in cell lysates (A). Transfected cells were treated with *C. perfringens* neuraminidase (NA) or mock-treated (PBS) for one hour at 37°C, stimulated with LPS (1 ng/ml) or no-stimulation (medium) for 16 h, and the lysates were evaluated for luciferase activity (B), and production of IL-8 (C). Transfected cells were mock-treated (PBS) or treated with NA alone, NA with specific inhibitor (NA+2-DN), or heat-inactivated NA (ΔNA), stimulated with LPS (1 ng/ml) for 16 h, and the lysates were evaluated for luciferase activity (D). Results shown are representative of data from at least 3 independent experiments, each with similar results. RLU (relative luciferase units) represent each sample's firefly vs. renilla luciferase activity.

Since we previously observed that bacterial NA treatment of intact human PBMC led to a synergistic increase in cytokine expression with LPS stimulation [16], we hypothesized that desialylation of the TLR4 complex contributed to the TLR4 signaling in response to bacterial LPS. We therefore utilized an established luciferase reporter gene transfection system [16] and studied the NFκB activation by luciferase activity detected in cell lysates. HEK293T cells alone, which lack endogenous TLR4 receptors, do not respond to LPS stimulation (data not shown). Following LPS stimulation, luciferase activity in TLR4/MD2/CD14-transfected cells increased in response to LPS dose-dependently (Figure 1A). Correspondingly, IL-8 production in culture supernatants increased and correlated to luciferase activities (data not shown).

To study the effect of NA treatment, the transfected cells were treated with NA before LPS stimulation. As shown in Figure 1B, the NA-treated transfected cells had a nearly 50% increase in the luciferase activity in response to LPS compared to those with no NA treatment (Figure 1B, p<0.05). IL-8 production also increased on LPS-stimulated cells with NA treatment (Figure 1C, p<0.05).

To exclude the possibility that the enhanced NFκB activation was due to additive activation by the impurities in the enzyme preparation, we treated the cells with NA in the presence of 2-DN—a neuraminidase inhibitor or with heat-inactivated NA. While increased from transfected cells with NA treatment, the luciferase activities from similarly treated cells in the presence of 2-DN is comparable to that from cells which did not receive NA treatment (Figure 1D). Heat-inactivated NA also failed to enhance the TLR4 signaling in response to LPS stimulation (Figure 1D). These results confirm that the enhancement in TLR4 signaling was dependent on the catalytic activity of NA which can be abolished by either inhibitor or heat inactivation.

### 2. Requirement of MD2 in TLR4 signaling

**Figure 2:**
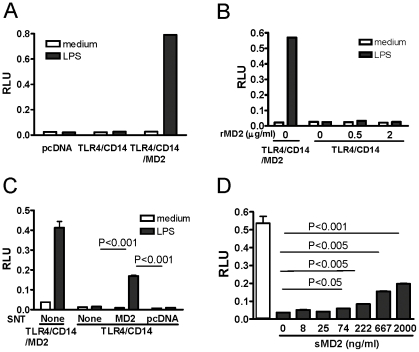
Requirement of MD2 in TLR4 signaling. HEK293T cells were transfected with a mixture of all plasmids as in Figure 1 (TLR4/CD14/MD2), plasmids encoding only TLR4 and CD14 (TLR4/CD14), or control plasmid (pcDNA), stimulated with LPS (1 ng/ml) for 16 h and the lysates were evaluated for luciferase activity (A). TLR4/CD14-transfected HEK293T cells were stimulated with LPS in culture in the presence of increasing amount of recombinant human MD2, and the lysates were evaluated for luciferase activity (B). Culture supernatants (SNT) from MD2- or empty vector- (pcDNA) transfected cells were added to TLR4/CD14-transfected cells prior to LPS stimulation and luciferase activity was determined in cell lysates (C). A specific amount of affinity-purified sMD2 was added in culture media of TLR4/CD14-transfected cells before LPS stimulation (dark bars). TLR4/CD14/MD2-transfected cells with LPS stimulation were included as positive control (open bars) (D). Results shown are representative of data from at least 3 independent experiments, each with similar results.

The TLR4 receptor complex consists of three critical components, TLR4, MD2, and CD14, for its optimal signaling. When HEK293T cells were transfected with the plasmid mixture without MD2, no luciferase activity was detected after LPS stimulation (Figure 2A). Since it was reported that the soluble form of MD2 (sMD2) supported LPS-mediated TLR4 signaling by recruiting LPS to the TLR4 complex and activated the downstream signaling [6], [23], we tested if we could supplement sMD2 and restore the responses of TLR4/CD14-transfected cells to LPS. *E. coli*-derived recombinant human MD2 (rhMD2, R&D) was added to the culture of TLR4/CD14-transfected cells before LPS stimulation. Over a wide range of concentrations (up to 2 µg/ml), the rhMD2 did not restore the luciferase activity in lysates of TLR4/CD14-transfected cells with LPS stimulation (Figure 2B). This finding was consistent with previous reports that MD2 was highly-glycosylated [6], [26] and that mutations at glycosylation sites abolished the TLR4 signaling [6], [26]. Since commercially available rhMD2 is made in *E*. coli, it is deficient in glycosylation.

Supernatant from MD2-transfected HEK293T cells was tested as a source of sMD2 to overcome the problem with bacterial-expressed MD-2. TLR4/CD14-transfected cells were supplemented with sMD2 before LPS stimulation. A significant amount of luciferase activity was detected, even though it was lower than that of TLR4/MD2/CD14-transfected cells (Figure 2C). As a control, little luciferase activity was detected with supplementation of supernatant from mock-transfected cells (pcDNA) (Figure 2C). Thus, our data confirmed that addition of sMD2 partially restored TLR4 signaling in reconstituted culture, and suggested that, like for TLR4 itself, appropriate glycosylation of MD2 was required for TLR4 signaling.

Although we were able to restore LPS-mediated signaling with addition of MD2-containing supernatant, the strength of that signal never approached that of the TLR4/MD2/CD14-transfected cells. Luciferase activity did not change using increasing amounts of MD2-containing supernatant in a wide range (data not shown) suggesting that the concentration of MD2 was not the limiting factor for optimal NFκB activation in the reconstituted culture. Indeed, it has been reported that picomolar amounts of sMD2 is sufficient to induce LPS-mediated TLR4 complex activation [23]. To further address this question, we purified sMD2 from the culture supernatant of MD2-transfected cells using an anti-FLAG affinity column. Again, supplementation of up to 2 ug/ml purified MD2 into TLR4/CD14-transfected cells restored the LPS-induced NFκB activation partially (Figure 2D). This result suggests that exogenous sMD2 could restore the TLR4 signaling.

### 3. Effect of mutations at glycosylation sites on the ability of TLR4 to mediate LPS-induced activation

**Figure 3:**
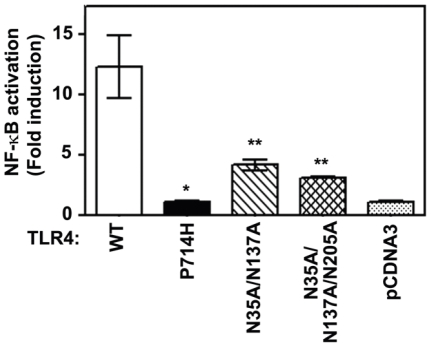
Effect of P714H, N35A/N173A, and N35A/N173A/N205A mutations on the ability of TLR4 to mediate LPS-induced activation. HEK293T cells were co-transfected with expression vectors encoding wild type TLR4 (WT) or P714H, N35A/N173A, and N35A/N173A/N205A mutants along with pEFBOS-MD2, pCDNA3-CD14, pELAM-luc, and pTK-*Renilla*-luc. Transfected cells were stimulated with LPS for 6 h, and firefly vs. renilla luciferase activities were measured in cell lysates. Data were processed using Student t-test. *p<0.005; **p<0.05 (vs. WT).

The TLR4 ectodomain contains nine N-linked glycosylation sites which are critical for TLR4 receptor function [6]. To examine the importance of glycosylation for TLR4 signaling, we used site-directed mutagenesis to introduce alanine substitutions of asparagines (N) at positions 35, 173, and 205 into TLR4-YFP. These sites were chosen since compromised LPS signaling exerted by these mutations could not be attributed to reductions in their expression levels compared to wild-type TLR4 [6]. To test the impact of these mutations on TLR4 signaling, CD14/MD2 co-receptors were overexpressed in HEK293T cells along with either wild-type or mutant TLR4 versions, and LPS-mediated activation of co-transfected NF-κB luciferase reporter was determined. We found that the N35A/N173A double glycosylation mutants exhibited LPS-mediated NF-κB reporter activation reduced by ∼75%, and the triple mutant N35A/N173A/N205A exerted ∼83% reduction (Figure 3). Mutagenesis of single N-linked glycosylation sites did not affect LPS-mediated NF-κB activation (data not shown and [6]), while P714H TLR4-YFP, which was made to mimic the non-signaling P712H mutation responsible for LPS unresponsiveness of C3H/HeJ mice, showed complete signaling deficiency (Figure 3A). These data indicate the importance of N-linked glycosylation for TLR4 signaling capacity.

### 4. Sialylation of TLR4 and MD2

**Figure 4:**
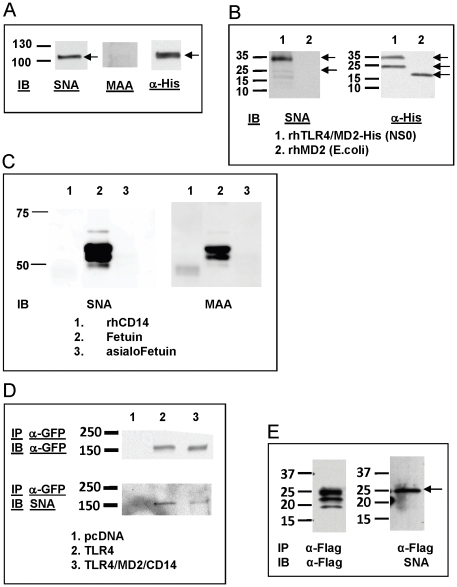
Sialylation of TLR4 and MD2. Recombinant human TLR4-His/MD2-His proteins expressed in the mammalian NS0 cell line were separated by SDS-PAGE and analyzed on immunoblot for binding to lectin SNA, MAAII and to anti-His antibody (A). Recombinant human TLR4-His/MD2-His expressed in NS0 cells (lane 1) or recombinant human MD2-His expressed in *E. coli* (lane 2) were separated by SDS-PAGE and probed by lectin blot with SNA (left) and on immunoblot with anti-His antibody (right) (B). Recombinant human CD14 (lane 1) were separated by SDS-PAGE and probed by lectin blot with SNA (left) and MAAII (right). The highly sialylated glycoprotein, fetuin (lane 2) and asialofetuin (lane 3) were included as positive and negative controls respectively (C). HEK293T cells were transfected with control pcDNA, or plasmids encoding TLR4-YFP alone or with MD2 and CD14 expression plasmids, and proteins from cell lysates were immunoprecipitated with ant-GFP antibody and probed on immunoblot with either anti-GFP antibody (top) or SNA (bottom) (D). Proteins from the medium of MD2-transfected cells were immunoprecipitated with anti-FLAG antibody and probed on immunoblot with anti-FLAG antibody (left) or SNA (right) (E). The expected molecular weights of MD2 and TLR4 are indicated by arrows. Results shown are representative of data from at least 2 independent experiments, each with similar results.

Even though it was known that TLR4 was glycosylated and N-linked glycosylation was important in its signaling, the glycan moiety is still unknown. Since our previous studies [16] suggested that TLR4 complex may contain sialic acid, we determined whether TLR4 and MD2 were sialylated using lectin blot technique established in our laboratory [14]. Lectins are a group of proteins that bind specifically to different glycoconjugates. SNA and MAAII recognize terminal sialic acid in α-2,6- or α-2,3-linkage respectively. The His-tagged recombinant human TLR4/MD2 mixture expressed in mammalian NS0 cells (R&D) was separated on SDS-PAGE, and blotted with SNA and MAAII. One band at 105 KD was identified (Figure 4A) with SNA, which corresponds to the reported size of recombinant TLR4. Anti-His antibody recognized the same size band in Western blot, but no band in this range was detected with MAAII blotting (Figure 4A).

A band at 34 KD was also revealed with SNA blot (Figure 4B) but was not observed in the lane with the rhMD2 expressed from *E. coli*. Anti-His antibody recognized a same size and another lower MW (25 KD) band in Western blot, perhaps two variant forms of MD2 (Figure 4B) previously observed [6]. No band in this range was detected with MAAII blotting (data not shown). Thus, our data confirm that both recombinant TLR4 and MD2 expressed in mammalian NS0 cells contained α-2,6-linked sialic acid residues.

CD14 is known to be glycosylated, but the glycan species have not been identified [27] [28]. We therefore examined the sialylation status of recombinant soluble human (rh) CD14 (R&D). Using the lectin blot technique we observed a weak but discernible band with MAAII but not with SNA, which indicates that CD14 is sialylated with α-2,3 glycosidic linkage (Figure 4C). Fetuin and asialofetuin were used as positive and negative controls respectively for the lectins.

To verify the TLR4-YFP in our transfected HEK293T cells was sialylated, we immunoprecipitated the TLR4 complex from the lysate of transfected cells with anti-GFP antibody and separated the protein on SDS-PAGE gel. The TLR4-YFP fusion protein was first located by blotting with anti-GFP antibody. A major band at 150 KD was identified from TLR4- or TLR4/MD2/CD14-transfected cell lysate but was absent from pcDNA-transfected cells. (Figure 4D, top panel). When blotted with SNA, the same size band at 150 KD was recognized (Figure 4D, bottom). These data suggested the TLR4-YFP from cell lysates was also sialylated with α-2,6-linkage.

Similarly, MD2-FLAG was immunoprecipitated from the culture supernatants of MD2-transfected cells with anti-FLAG antibody and separated on SDS-PAGE. Three bands at 15–25 KD were identified (Figure 4E, left) when blotting with anti-FLAG antibody, which corresponds to three variations in glycosylation previously reported [6]. When blotted with SNA, one band at 25 KD was identified (Figure 4E, right). No band in this range was detected with MAAII blotting (data not shown). These data suggest that at least one isoform of glycosylated MD2 contained terminal sialic acid with α-2,6-linkage.

### 5. Optimal signaling when both TLR4/CD14-transfected cells and sMD2 are treated with neuraminidase

**Figure 5:**
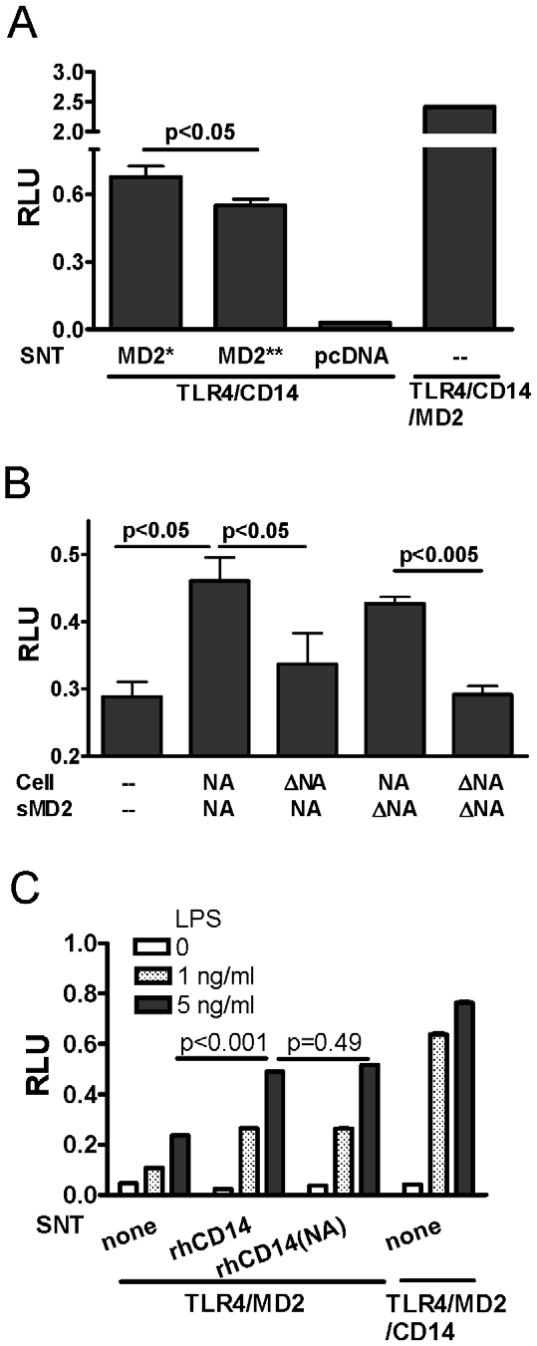
Optimal LPS-induced signaling when both TLR4/CD14 and MD2 treated with neuraminidase. HEK293T cells that were transfected as in Figure 2 and TLR4/CD14-transfected cells were supplemented with NA-treated SNT from MD2 transfected cells (MD2*), heated-inactivated NA-treated SNT (MD2**), or SNT from empty vector transfected cells (pcDNA) before LPS stimulation (5 ng/ml). Cell lysates were harvested after 16 h for reporter assay (A). TLR4/CD14-transfected HEK293T cells were treated with NA, heat-inactivated NA (ΔNA), or were untreated (-) for one hour at 37°C, stimulated with LPS (5 ng/ml) for 16 h in the presence of SNT from MD2 transfected cells (sMD2), which had been pre-treated with NA agarose (NA), heated-inactivated NA agarose (ΔNA), or were untreated, and luciferase activities were determined in cell lysates (B). TLR4/MD2-transfected HEK293T cells were supplemented with NA treated recombinant human CD14 (rhCD14[NA]), untreated CD14 (rhCD14), or medium (none) prior to LPS stimulation (1–5 ng/ml). Cell lysates were harvested after 16 h for reporter assay (C). Results shown are representative of data from at least 3 independent experiments, each with similar results.

As removal of sialyl residues from the transfected cells enhanced LPS-mediated signaling, we determined whether desialylation of either or both TLR4/CD14 on cells and MD2 contributed to the signaling enhancement. We treated sMD2 with NA and added it into the above reconstituted culture. Heat-inactivated NA was used to treat supernatant as a control. Addition of desialylated sMD2 restored a moderate but significant NFκB signal unlike the case with the MD2 treated with heat-inactivated NA (Figure 5A, p = 0.036). This result suggested that desialylation of sMD2 enhanced LPS-induced TLR4 signaling.

To determine whether TLR4/CD14 desialylation contributes to the signaling enhancement, we treated TLR4/CD14-transfected cells with NA or heat-inactivated NA. After sMD2 reconstitution, NA-treated cells had stronger NFκB activation than untreated cells after sMD2 reconstitution. Given these findings we then asked whether optimal signaling could be obtained with both TLR4/CD14 on the cells and sMD2 treated with NA before reconstitution. As anticipated, the highest NFκB activation was observed when both cells and supernatant were treated with NA (the second histogram in Figure 5B); suboptimal enhancement was observed with desialylation of either TLR4/CD14-bearing cells (the fourth histogram) or MD2-containing supernatant (the third histogram in Figure 5B). Desialylation of cells had a better result than desialylation of sMD2. The combined results suggested that desialylation of both is needed for optimal signaling. The luciferase activity following LPS treatment of both TLR4/CD14-transfected cells and sMD2, each treated with heat-inactivated NA, was similar to that of cells and sMD2 not exposed to NA (Figure 5B, fifth histogram vs. first histogram).

Since we demonstrated that desialylation of HEK293 cells transfected with plasmids encoding TLR4 and CD14 resulted in a marked increase in luciferase activity (Figure 5B), we next dissected the relative contribution of the TLR4 and CD14 components, each of which is sialylated, to the NA-enhanced signal. CD14 may be present either as a membrane-bound protein on the surface of cells, or as a circulating, soluble protein [28], [29]. HEK293T cells were transfected with TLR4/MD2 (*i.e.* in the absence of CD14) and supplemented with soluble, rhCD14 that was treated with or without NA before addition to the culture prior to LPS stimulation for luciferase assay. TLR4/MD2- transfected cells exhibited decreased luciferase activity in the absence of exogenous CD14 (Figure 5C). The minimal response in the absence of transfection of cells with the CD14-encoding plasmid may be due to CD14 that was present in the culture medium, as previously reported [21].. Addition of rhCD14 further increased the luciferase activity from TLR4/MD2-transfected cells (Figure 5C); however, there was no significant difference between the addition of NA-treated and untreated rhCD14 (Figure 5C) or with heat-inactivated NA-treated CD14 (data not shown). This suggests that the moderate level of sialylation on CD14 plays no role in the NA-enhanced signaling through TLR4.

### 6. Dimerization of TLR4 receptor with LPS stimulation

**Figure 6:**
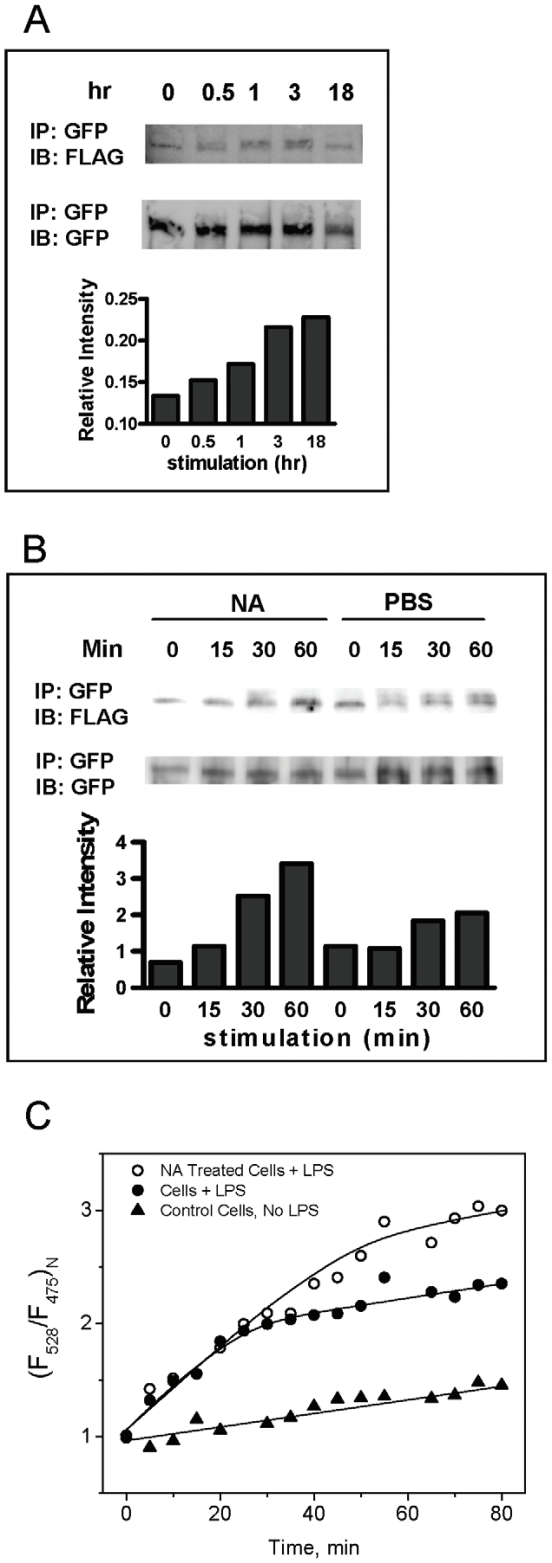
NA treatment of TLR4-expressing HEK293T cells enhanced TLR4 dimerization. HEK293T cells were transfected with mixture of expression vectors encoding TLR4-YFP, TLR4-FLAG, MD2-HA, and CD14 for two days. At indicated time points after stimulation with LPS (10 µg/ml), cell lysates were harvested and proteins were immunoprecipitated with anti-GFP antibody, separated on SDS-PAGE gels and probed on immunoblot with either anti-FLAG or anti-GFP antibodies to reveal the individual tagged TLR4 protein (A). Transfected cells were treated with either NA or PBS (as control) for 1 hour before LPS stimulation and processed as above (B). The captured images were analyzed using ImageJ (NIH, USA) and the relative intensities were obtained by dividing the mean intensity of the bands on Western blot using anti-FLAG antibody over that using anti-GFP blot. HEK293T cells were transfected with mixture of plasmids encoding TLR4-CFP, TLR4-YFP, MD2-HA, and CD14 and were maintained in culture for two days, re-suspended, and treated with NA (30 mU/ml, open circles) or PBS (filled symbols) for 1 hr. Cells were transferred to a cuvette, where they were stimulated with LPS (10 µg/ml, circles) or control PBS (triangles). The fluorescence spectrum for each sample was recorded at different time points, and the F528/F475 ratio was plotted (C). Results shown are representative of data from at least 3 independent experiments.

During LPS stimulation, dimerization of TLR4 must occur (including the interaction with MD2-LPS) for MyD88 recruitment and triggering of downstream signaling [30], [31]. We speculated that desialylation might facilitate TLR4 dimerization and therefore enhance LPS-induced TLR4 signaling. To address this question, we co-transfected HEK293T cells with YFP- and FLAG-tagged TLR4 plasmids along with CD14 and MD2 and assayed for the interaction of the two tagged TLR4 molecules by immunoprecipitating YFP-tagged TLR4 from lysates of tranfected cells (by anti-GFP antibody) and immunoblotting the FLAG-tagged protein . A small fraction of FLAG-tagged protein could be detected after GFP immunoprecipitation in the absence of LPS stimulation, which increased after 30 minutes of LPS stimulation (Figure 6A). This time-dependent increase in co-immunoprecipitation suggested that LPS stimulation increased the dimerization of two tagged TLR4.

To study the effect of NA treatment in LPS-induced TLR4 dimerization, transfected cells were treated with NA (30 mU/ml) or PBS (untreated control) for 1 h prior to LPS stimulation. Compared to the untreated group (PBS), earlier dimerization was seen in the NA-treated cells at 15 minutes and the TLR4 dimerization was greater at 60 minutes after LPS stimulation (Figure 6B).

FRET analysis using TLR4 protein tagged with fluorescent proteins (CFP and YFP) at the C-terminal end further confirmed that TLR4 dimerization occurred and was enhanced with NA treatment. HEK293T cells were transfected with plasmids encoding TLR4-YFP, TLR4-CFP, MD2, and CD14 and stimulated with LPS. Formation of CFP- and YFP-tagged TLR4 heterodimers was then measured using FRET (Supporting information S1, Figure S1). After LPS addition, the FRET signal from CFP/YFP cells exponentially increased and reached saturation (equilibrium) at >20 min (Figure 6C), while cells without LPS stimulation (control) showed only a slight non-specific increase in FRET ratio with time. The observed exponential change in FRET signal exhibited low-rate kinetics (characteristic time of kinetics is about 20 min) of TLR4 dimerization, which could be explained by slow lateral diffusion of TLR4 monomers within a viscous plasma membrane. It should be noted that the TLR4 dimer is a dynamic system which is in equilibrium with the monomers, with the state of equilibrium depending on the dimerization constant. The value of the FRET signal reflects not only the interaction between TLR4 monomers, but also the state of equilibrium, i.e. affinity of the proteins to each other. We observed a similar effect of the binding equilibrium on the asymptotic FRET value for protein/DNA interactions [32], [33].

The rates of TLR4 dimerizaton after LPS stimulation of NA treated cells and non-treated cells were similar, but the asymptotic value of the FRET signal for NA-treated cells was significantly greater as compared to non-treated cells. The increase in FRET signal for NA treated cells in a state of equilibrium, (t>40 min,) could be explained by the shift of equilibrium to dimers in the binding reaction, TLR4+TLR4?(TLR4)_2_, i.e. the affinity of NA-treated TLR4 proteins to each other is greater as compared to non-treated TLR4 proteins. It also assumes that NA treatment of cells could modify the structure (desialylation) of TLR4 and cause tighter contact between components of the dimer.

### 7. 2-DN inhibits TLR4 signaling

**Figure 7:**
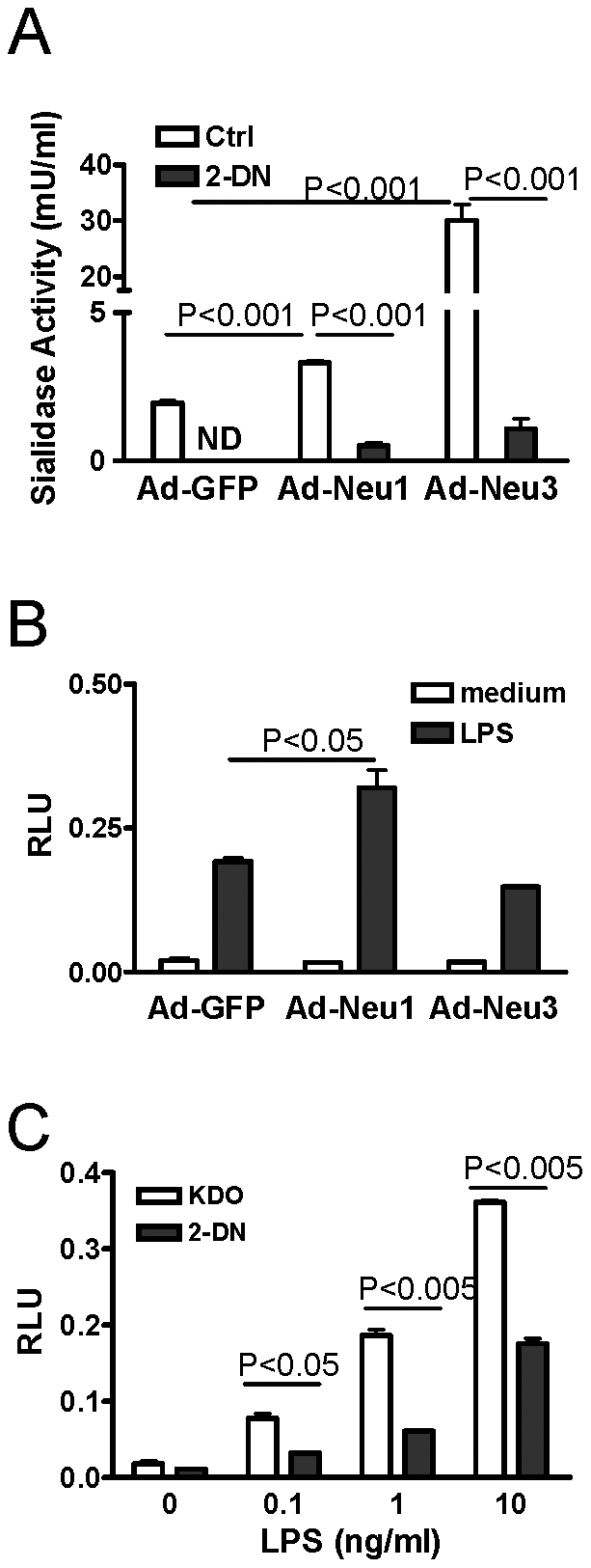
Inhibition of endogenous sialidase activity suppressed LPS-induced NFκB activation in HEK cells. HEK293T cells were infected with recombinant adenoviruses expressing human Neu1 (Ad-Neu1) or Neu3 (Ad-Neu3), or with virus containing empty vector (Ad-GFP) and sialidase activity from cell lysates was assayed using 4-MUNANA as substrate in the absence or presence of the sialidase inhibitor 2-DN (250 µg/ml) (A). TLR4/CD14/MD2-transfected HEK293T cells were further infected with Ad-Neu1, Ad-Neu3 or Ad-GFP, for 2 days, stimulated with LPS (1 ng/ml) for 16 h, and evaluated for luciferase activity (B). TLR4/CD14/MD2-transfected HEK293T cells were incubated with 2-DN (250 µg/ml) or KDO (250 µg/ml) for 2 days, and stimulated with different concentrations of LPS for 16 h prior to analysis of cell lysates for luciferase activity (C). Results shown are representative of data from at least 3 independent experiments, each with similar results. ND: not done.

Mammalian sialidases share homolog and functional similarity with bacterial or viral neuraminidases [34], [35]. We speculated that desialylation by endogenous human sialidases affects TLR4 signaling similar to what we observed with bacterial neuraminidase demonstrated above. Four human sialidases had been identified, with particular cellular localizations, and perhaps different cellular functions [35]. Lysosomal sialidase (Neu1) is the most abundant sialidase which associates with other proteins to form a multienzyme complex in lysosomes [36]. It is also expressed on the surface of diverse types of cells [37]–[40]. Membrane-associated sialidase (Neu3) preferentially desialylates gangliosides and perhaps many surface glycoproteins [41], [42]. To confirm the ability of 2-DN to inhibit sialidase, we first overexpressed Neu1 and Neu3 with contructs of recombinant adenovirus expressing human Neu1 (Ad-Neu1) or Neu3 (Ad-Neu3). To verify the sialidase activities, we performed a 4-MUNANA assay after HEK293T cells were infected with recombinant adenovirus. Compared to a mock control (Ad-GFP), both Ad-Neu1 and Ad-Neu3 infected cells had significantly higher enzymatic activities, which were decreased by the neuraminidase inhibitor 2-DN at least 10-fold (Figure 7A).

To test the effect of overexpression of sialidase on LPS-induced TLR4 signalling, the TLR4/CD14/MD2-transfected cells were infected with various recombinant adenoviruses and stimulated with LPS. Compared to Ad-GFP, Ad-Neu1, but not Ad-Neu3 infected cells had higher NFκB activation in response to LPS stimulation (Figure 7B). Therefore, overexpression of human Neu1 enhanced cell responses to LPS.

Since HEK293T cells also demonstrated endogenous sialidase activity (Figure 7A), we speculated that the neuraminidase inhibitor, 2-DN, would suppress the desialylation of the TLR4 complex by endogenous sialidase and therefore decrease the cells' response to LPS. The TLR4/MD2/CD14-transfected cells were incubated with 2-DN or KDO (as mock control) before and during LPS stimulation. The presence of 2-DN decreased the luciferase activities in the lysates of the transfected cells in response to LPS stimulation significantly (Figure 7C). This suggested that desialylation by endogenous sialidase also optimized TLR4 signaling in the same manner as bacterial NA and inhibition of sialidase activities lowered LPS-induced NFκB activation.

## Discussion

Our earlier finding that NA pre-treatment of human PBMCs enhanced their pro-inflammatory cytokine response following a subsequent stimulation with LPS suggested that the TLR4 receptor complex was sialylated, and that modulation of sialyl residues on the TLR4 might affect TLR4-mediated LPS signaling [16]. In the present study we show that two components of the TLR4 complex, TLR4 and MD2, have sialyl residues in an α-2,6 glycosidic linkage and that NA treatment of both MD2 and TLR4 independently and additively enhance TLR4 complex-initiated signaling. Human CD14 was weakly sialylated in a α-2,3 glycosidic linkage. LPS-induced NFκB activation was increased with overexpression of human cytoplasmic sialidase (Neu1) and endogenous sialidase activity was suppressed with 2-DN, suggesting a role for endogenous HEK293T cell sialidase in optimal TLR4 signaling. Thus, modulation of the sialyl status of these molecules may represent an additional mechanism by which their activity is regulated.

Using a previously described experimental system in which HEK293T cells that lack TLR4 are transfected with plasmids encoding TLR4, MD2 and CD14, we were able to recapitulate the requirements for LPS signaling. Using either a luciferase NFκB dependent reporter system or IL-8 expression to monitor the response, we found that when transfected with TLR4/CD14/MD2, LPS treatment induced these cells to respond, and that NA pretreatment increased this response. This increased response was abrogated either by addition of a NA inhibitor, 2 DN, or heat inactivation of NA. Our reconstituted HEK293T experimental system was also capable of demonstrating the importance of glycosylated MD2.

Using endoglycosidase treatment and site-directed mutagenesis of N-linked glycosylation sites, Correia and Ulevitch reported that N-linked glycosylation sites on both MD2 and TLR4 were essential LPS signaling [6], but did not identify the glycans that were important or suggest whether modification of these glycosylation sites might contribute to the regulation of their response. In our studies, when the plasmid for MD2 was omitted from the transfection of HEK293T cells, LPS was unable to induce a luciferase response, as previously observed by Visentin et al [43]. Supplementation of the TLR4/CD14-transfected HEK293T cell cultures with a non-glycosylated rMD2 made in prokaryotic cells was unable to restore the activity (Figure 2B); however, addition of culture supernatants from MD-2 transfected cells or affinity purified protein from these supernatants that were each glycosylated reconstituted HEK293T cell responsiveness to LPS . Picomolar amounts of ectopically-tagged sMD2 from transfected HEK293T cells were reported to activate TLR4 [23]. Therefore, glycosylation of MD2 is required for TLR4 signaling.

MD2 was originally discovered by its sequence homology with MD-1, a protein associated with a B cell homologue of TLR4, RP 105 [44]. It is an Ig domain-folded protein with a deep hydrophobic pocket. CD14 is a membrane-bound protein expressed on myeloid cells and is present in plasma as a soluble protein. It functions as a soluble LPS receptor that recognizes LPS bound to LPS binding protein- and transfers the monomeric LPS to MD2 which then binds to TLR4 [28]. Thus, MD2 bridges the recognition of LPS by LBP and CD14 to the activation of TLR4 through the high-affinity, non-covalent binding to the N-terminal ectodomain of TLR4 [45]. Soluble MD2 (sMD2) in supernatants of MD2-transfected HEK293T cells represents a heterogenous collection of unstable and mainly inactive oligomers; however, only the MD2 monomer is capable of binding the LPS and activating TLR4 [45], [46].

Exogenous soluble MD2 was not able to fully reconstitute the LPS responsiveness that we measured when HEK293T cells were transfected with TLR4/MD2 /CD14. Earlier studies suggested that MD2 facilitates the expression of TLR4 on the cell surface [47]. Thus the lower response to the addition of sMD2 could be attributed to the reduced expression of TLR4 on the HEK293T surface, although another study challenges that conclusion [48]. Alternatively, since sMD2 exists primarily as inactive, unstable multimers, there might not have been a sufficient number of MD2 monomers added to provide the required activity. Even when added as affinity-purified protein from the culture supernatants, we observed a plateau of responsiveness with increasing amounts of MD2 protein which suggests that the problem could have been the activity of the sMD2 and not the amount of protein in the supernatant.

We also show that N-linked glycosylation of TLR4 is required for TLR4-mediated signaling and that TLR4 has α-2,6 sialyl residues. Since it is not possible to desialylate only TLR4 on primary cells without affecting other cell surface molecules, we could not directly prove the effect of desialylation of TLR4; however, several lines of evidence support this conclusion. First, desialylating cells without MD2, one component of the TLR4 complex, did not induce any signaling by LPS, which suggests that it is TLR4-mediated signaling (data not shown). Secondly, desialylation of cells alone, without LPS stimulation, induced only minor signaling. Thirdly, desialylation increased TLR4 dimerization which is required to initiate its downstream signaling cascade. When the TLR4/CD14 complex and MD2 in HEK293T cell cultures were independently treated with NA, removal of sialyl residues from each of these components contributed to the optimally enhanced LPS responsiveness (Figure 5) with the sialic acid modulation of TLR4/CD14 having a more critical role than desialylation of sMD2. The lesser effect of desialylation on sMD2 may be attributable in part to our finding (Figure 4D) that only one of the three previously described protein isoforms of MD2 is recognized by the lectin, SNA. Thus, it is possible that in addition to its α-2,6 sialyl glycoform, MD2 may have additional glycosyl species critical to LPS-mediated signaling. We are investigating this possibility in our laboratory.

Human CD14 is predicted to have four N-linked glycosylation sites [49], [27] as well as O-linked glycans [28]. The glycan species that may be associated with CD14 have yet to be identified. We find that rsCD14 has an α-2,3-linked sialyl residue that does not appear to contribute to the NA-enhanced LPS-initiated signaling through TLR4 (Fig 5C). This suggests that, like MD-2, other glycans may play a more important role in contribution to the structure and function of CD14.

The seeming contradiction that N-linked glycosylation of TLR4 and MD2 is required for their functional activity, yet removal of sialyl residues enhances their activity can be explained by the fact that sialyl residues often are present at a terminal position on glycans. Removal of these terminal sialyl residues by sialidases (or the addition by sialyltransferases) may rapidly modulate the charge or conformation of these glycans and affect their functional activity. Thus while glycans are critical to TLR4 complex function, modification of sialyl residues on those glycans provide an additional measure of regulation. Further, it is likely that the removal of terminal sialyl residues exposes subterminal galactose residues on these glycans which may then serve as targets for galectins that may have either pro- or anti-inflammatory properties [50].

Appropriate glycosylation of proteins might be crucial for the interaction of LPS with elements of TLR4 complex. It has been suggested [48] that the interaction of MD2 and TLR4 may induce a conformational change that leads to the dimerization required for signaling. Our data suggest that the terminal sialic acids on the glycan may have a negative effect on TLR4 signaling. With its negative charge, sialic acid might hinder the interaction between TLR4 monomers or the MD2-LPS interaction with the TLR4 ectodomain. Removal of the negatively charged sialic acid on the molecule surface, may facilitate MD2-TLR4 interaction and/or TLR4-TLR4 dimerization (Figure 6B, C). In the absence of identifying the size and nature of the glycan structures on TLR4, it would be difficult to predict how glycosylation might affect the published structure of this molecule and its interaction with MD-2 and ligand [51], [52].

LPS is believed to bind directly to MD2, and this LPS/MD2 complex binds to the TLR4 ectodomain which results in TLR4 dimerization and ultimately intracellular signaling. In the current study we did not evaluate whether removal of sialyl residues of MD2 enhanced the binding of LPS to MD2; however, MD2 is unstable in the absence of LPS binding [53]. We now show by both co-immunoprecipitation experiments and FRET analysis that LPS treatment initiates TLR4 dimerization and that NA pretreatment facilitates this interaction.

Finally we suggest that human sialidase is capable of mediating these NA-enhanced responses. While HEK293T cells have some sialidase activity (Figure 7A), co-expression of Neu1 and 3 with the TLR4 complex resulted in increased HEK293T cells sialidase activity that was inhibitible by 2DN, the sialidase inhibitor (Figure 7B, C). In the absence of exogenous NA, LPS treatment induced a luciferase response that was inhibitable by 2DN, but not KDO. Thus, either Neu1 or Neu3 or an endogenous HEK293T sialidase (Figure 7C) could be a source of the sialic acid modulation in the TLR4 receptor complex. We previously showed in human PMNs that upon stimulation, sialidase activity was mobilized from secondary granules within the cell to the plasma membrane where it became an integral plasma membrane protein capable of removing sialyl residues from glycoconjugates on its own (autocrine) or adjacent (paracrine) cell surfaces [11], [12]. These studies were completed before the identification of the 4 *neu* isoforms. In a recently published study we were able to show that upon activation endogenous PMN sialidase activity could play a role in leukocyte trafficking by unmasking an activation epitope on β2 integrins and that Neu1 co-localized with the β2 integrin on fluorescence microscopy [14]. Further, the integrin binding partner, ICAM-1, on endothelial cells could be “activated” for enhanced β2 integrin binding by removal of sialyl residues from ICAM-1. Together, these data suggest that with cell activation, sialidase mobilization, perhaps Neu 1, could be a source of sialyl residue modification.

Since our initial observation that NA pretreatment of human PBMCs enhanced the responsiveness to LPS, Amith *et al* later reported that TLR4 was sialylated with a α-2,3 linkage and showed Neu1 colocalization with TLR4 by fluorescent microscopy [40], [54]. In neither study was the role of glycosylation of MD2 or CD14 examined. Our study differs from this report by observing that both MD2 and TLR4 are required for signaling and both are sialylated in an α-2,6, not an α-2,3 glycosidic linkage. Further, we demonstrate dimerization of TLR4 monomers is enhanced by removal of sialyl residues by two different methods (Figure 6).

These studies demonstrate that modification of protein glycosylation is a rapid, additional mechanism by which cells regulate the activity of their receptor and other surface glycoconjugates important to immune responses. Further, it is likely that microbial pathogens that express NA may take advantage of these regulatory mechanisms to subvert the host immune response as part of their virulence strategy. This possibility offers a rich opportunity for further investigation and therapeutic targeting.

## Supporting Information

Figure S1 **Dimerization of TLR4 receptors can be effectively measured using the FRET approach.** (A) The fluorescence spectra of HEK293T cells transfected with TLR4-YFP (line 1). The background signal was measured using the control non-transfected cells (line 2). The fluorescence spectrum of the TLR4-YFP cells was corrected by subtracting the background signal (line3). Fluorescence was excited at 490 nm. (B) The fluorescence spectra of HEK293T cells, transfected with either TLR4-YFP, TLR4-CFP, or both TLR4-YFP and TLR4-CFP, showed the characteristic FRET change with time after LPS stimulation. With excitation at 440 nm, the fluorescence spectra of CFP and YFP were spectrally separated: CFP emission had maximum at 475 nm and YFP at 528 nm. After LPS stimulation, excitation at 440 nm resulted in a YFP (acceptor) emission increase, indicating protein association.
(TIF)

Supporting Information S1 **Supporting methods.**
(DOC)
