# Supplementary figures and images for: Sialyl Residues Modulate LPS-Mediated Signaling through the Toll-Like Receptor 4 Complex
Source: PLoS One. 2012 Apr 9;7(4):e32359. doi: 10.1371/journal.pone.0032359 (PMC3322133; doi:10.1371/journal.pone.0032359)

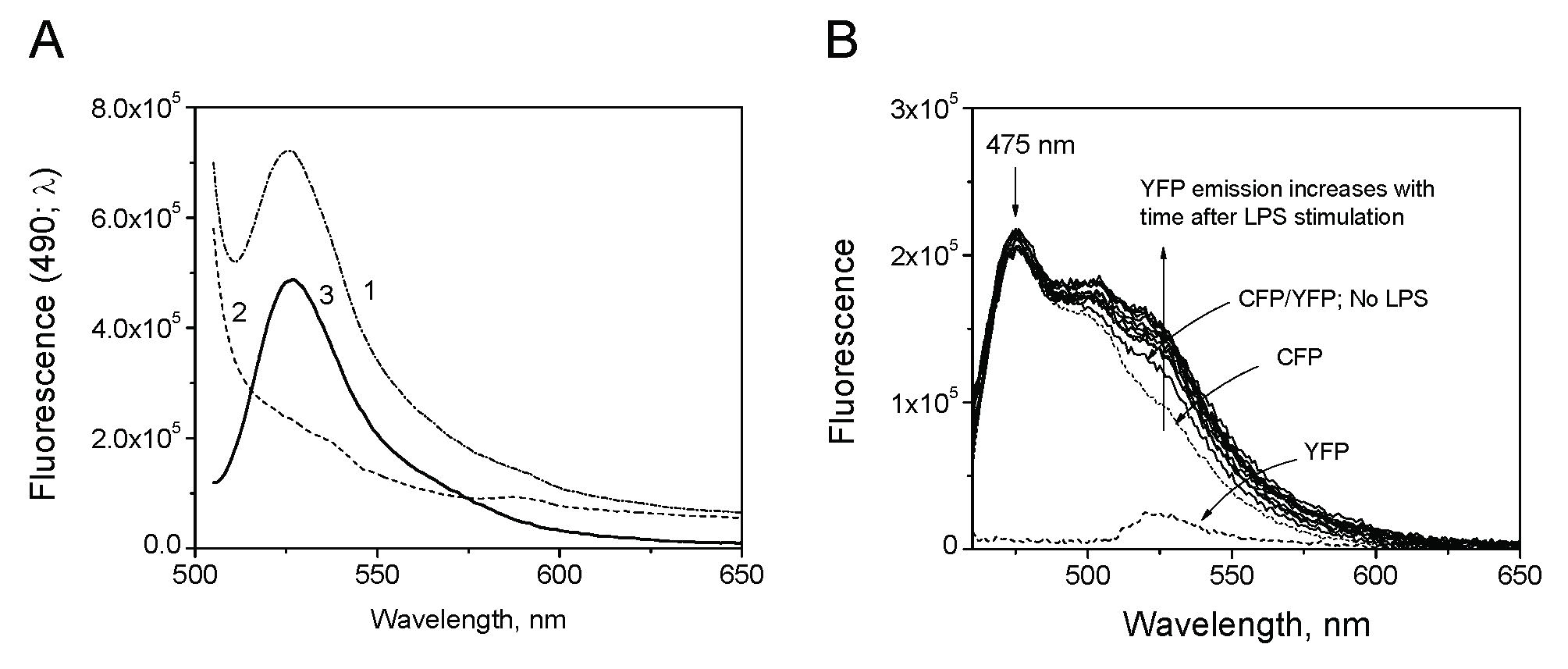

Supplement: Figure S1 — Dimerization of TLR4 receptors can be effectively measured using the FRET approach. (A) The fluorescence spectra of HEK293T cells transfected with TLR4-YFP (line 1). The background signal was measured using the control non-transfected cells (line 2). The fluorescence spectrum of the TLR4-YFP cells was corrected by subtracting the background signal (line3). Fluorescence was excited at 490 nm. (B) The fluorescence spectra of HEK293T cells, transfected with either TLR4-YFP, TLR4-CFP, or both TLR4-YFP and TLR4-CFP, showed the characteristic FRET change with time after LPS stimulation. With excitation at 440 nm, the fluorescence spectra of CFP and YFP were spectrally separated: CFP emission had maximum at 475 nm and YFP at 528 nm. After LPS stimulation, excitation at 440 nm resulted in a YFP (acceptor) emission increase, indicating protein association. (TIF) [file pone.0032359.s001.tif]
