# Supplementary material for: Sialyl Residues Modulate LPS-Mediated Signaling through the Toll-Like Receptor 4 Complex
Source: PLoS One. 2012 Apr 9;7(4):e32359. doi: 10.1371/journal.pone.0032359 (PMC3322133; doi:10.1371/journal.pone.0032359)
Supplement: Supporting Information S1 — Supporting methods. (DOC) [file pone.0032359.s002.doc]

**Supplementary information**

The fluorescence spectrum of the CFP protein overlaps the absorption spectrum of YFP which creates conditions for fluorescence resonance energy transfer (FRET) from the cyan (CFP) to yellow (YFP) fluorescent protein, and makes them donor-acceptor pair [24, 25] suitable for estimation the change in distance between receptors. The efficiency of FRET (E) is sensitive to the distance between the donor and acceptor (R_DA_), and depends on Förster distance (R_o_), i.e. the distance at which energy transfer efficiency is 50 %: R_DA_ = R_o_×[(1-E)/E]^-6^. For the CFP-YFP pair the Förster distance R_o_ = 49 Å [2].

In this study we have used FRET approach to analyze the LPS-stimulated association of the TLR-4 receptors. For that purpose receptor proteins were tagged with fluorescent proteins (CFP and YFP) to the C-terminal end of the protein and transfected into HEK293T cells. The emission spectra of the fluorescent proteins (CFP and YFP) in transfected cells was measured and recorded in the spectral range from 460 nm to 650 nm. To excite YFP protein fluorescence alone, we used the 490 nm wavelength laser, at which only YFP absorbs light. The contribution of light scattering to the fluorescence spectra of transfected cells (background) was measured using samples containing the original (not transfected) HEK293T cells and this was subtracted from the sample fluorescence signals (Figure S1A).

For the FRET experiments, fluorescence was excited at 440 nm wavelength, at which both fluorescent proteins (CFP and YFP) absorb light. Analysis of the fluorescence spectra of CFP/YFP cells showed no FRET between donor (CFP) and acceptor (YFP) dyes (Figure S1B) without LPS stimulation, which indicated that the distance between TLR4 proteins is large (>> 50 Å) and one can assume that they do not interact with each other. After addition of the stimulation factor, LPS, the FRET signal from CFP/YFP cells increased with time (Figure S1B), and indicated protein association. Modification of TLR4 with fluorescent proteins did not influence on the structural state of the receptors in cell.
